# *Macrotylushenryi*, a new species of *Pelargonium*-feeding Cremnorrhinina from South Africa (Hemiptera, Miridae, Phylinae, Cremnorrhinini)

**DOI:** 10.3897/zookeys.796.21429

**Published:** 2018-11-15

**Authors:** Ruth Salas, Randall T. Schuh

**Affiliations:** 1 Division of Invertebrate Zoology, American Museum of Natural History, New York, NY 10024, USA American Museum of Natural History New York United States of America

**Keywords:** Geraniaceae, Heteroptera, host plant, long free pulvilli, plant bug, Western Cape

## Abstract

*Macrotylushenryi* is described as a new species from South Africa. This new taxon is recorded as feeding on species of *Pelargonium* (Geraniaceae) in the Western Cape. Documentation is provided in the form of diagnosis, description, habitus photographs, scanning electron micrographs, illustrations and images of genitalic structures, detailed distributional data, host plant information, and images of hosts and habitats. Morphological traits are similar to species of *Macrotylus* Fieber from the Northern Hemisphere, but coloration is substantially variable, and the structure of the male genitalia is distinctive.

## Introduction

*Macrotylus* Fieber, 1858 is now placed in Cremnorrhinini: Cremnorrhinina based on phylogenetic analysis that includes morphology and DNA sequence data (Schuh and Menard 2013, Menard et al. 2014). The genus is widely distributed in the Palearctic and Nearctic regions, with approximately three quarters of the total of 70 species described from the Palearctic (Schuh 2002–2013). Only two species have been recorded and described from the Ethiopian region (Schuh 1974).

Schuh (1974) described *Macrotylushemizygiae* and *M.niger* from Limpopo, Mpumalanga, and KwaZulu-Natal, South Africa. He indicated that *Macrotylus* can be recognized in South Africa by the strongly anteriorly projecting clypeus, the long free pulvilli, and the absence of heavy setiform setae on the dorsum. Schuh (1974) also reported only one host plant, *Hemizygiathorncroftii* Ashby (Lamiaceae), for this genus in South Africa. No additional taxa have since been recorded from the Ethiopian region.

Collecting of Miridae in South Africa produced new specimens of *Macrotylus* as well as host and localities documentation. Fieldwork focused on Namaqualand, the Little Karoo, and the *fynbos* vegetation of the Western Cape, because of the extreme botanical diversity of the area and the very limited sampling of Miridae for the area during the eight-month expedition of J.A. Slater and colleagues during 1967–1968, which had been the only concerted effort to collect phytophagous Heteroptera in South Africa.

In the present paper a new species, *Macrotylushenryi*, is described which feeds on plant species in the family Geraniaceae endemic to South Africa. This new taxon is dedicated to Thomas J. Henry in recognition of his contributions to our knowledge and understanding of true bug taxonomy and host associations.

## Materials and methods

Unique matrix code labels were affixed to each of 360 specimens examined; these codes are therefore referred to as “unique specimen identifiers” (USIs). The USI codes are composed of an institution and project code (AMNH_PBI) and a unique number (00393079). The AMNH_PBI prefix was removed from the Specimens Examined sections of the paper to save space and make these sections more readable, but was retained for the holotype and figures. Data for these specimens were captured using the American Museum of Natural History instance of the Arthropod Easy Capture specimen database, formerly known as Planetary Biodiversity Inventories database. Specimen data can be viewed on line through research.amnh.org/pbi/heteropteraspeciespage/ and discoverlife.org, and through the iDigBio web portal (idigbio.org/portal).

Color digital habitus images of the bugs were prepared using a Microptics-USA/Visionary Digital photomicrographic system as developed by Roy Larimer; multiple layers were stacked using Helicon Focus. Habitus photos are proportional to the size of the actual specimens so that relative sizes can be deduced from comparison of the specimen images.

Details on specimen measurements are provided in Table 1. All measurements are in millimeters, and were made using a micrometer driven stage, micrometer output being written directly to a spreadsheet. Summarized measurements were prepared with Excel for Windows (Microsoft Office 2013 Professional).

Host samples were collected and pressed in the field in conjunction with each collecting event. These specimens were subsequently identified by botanical specialists; the botanical names were then associated with the individual bug specimens through a specimen database and during the labeling process. *Macrotylus* specimens were collected on plants in flower. Thus, the identification of the host plants is to the level of species. Host field photos were made using a Nikon D1 SLR digital camera.

Scanning electron micrographs were prepared using a Hitachi S-4700 digital SEM. Male genitalic illustrations were prepared as pencil drawings using a Nikon Eclipse 80i compound microscope, then scanned and rendered as graphics using Adobe Illustrator. All such illustrations were drawn with a 20× or 40× objective lens. Female genitalic images were taken with a 10× or 20× objective lens using a Nikon E800 compound microscope, photomicrographic attachment, and software.

The insect specimens examined in this study were provided by the following institutions, or material is deposited in them; institutional abbreviations used in the specimens examined sections and names of individuals who assisted handling the specimens are also listed:

**AM**Australian Museum, Sydney, Australia; G. Cassis, D. Britton, D. Smith

**AMNH** American Museum of Natural History, New York

**CNC**Canadian National Insect Collection, Agriculture Canada, Ottawa; M.D. Schwartz, R.G. Foottit

**PPRI**Plant Protection Research Institute, Pretoria, South Africa; I.M. Millar

**SAMC**Iziko (South African) Museum, Cape Town, South Africa; S. van Noort

**UNSW**University of New South Wales, Australia; G. Cassis

**USNM**United States National Museum of Natural History, Smithsonian Institution, Washington, D.C.; T.J. Henry

**ZISP**Zoological Institute, Russian Academy of Sciences, St. Petersburg; F. Konstantinov, D. Gapon

## Taxonomy

### Macrotylus henryi sp. n.

Taxon classificationAnimaliaHemipteraMiridae

http://zoobank.org/0EBCA72F-0686-4894-B777-2179891A4754

**Figure 1.**
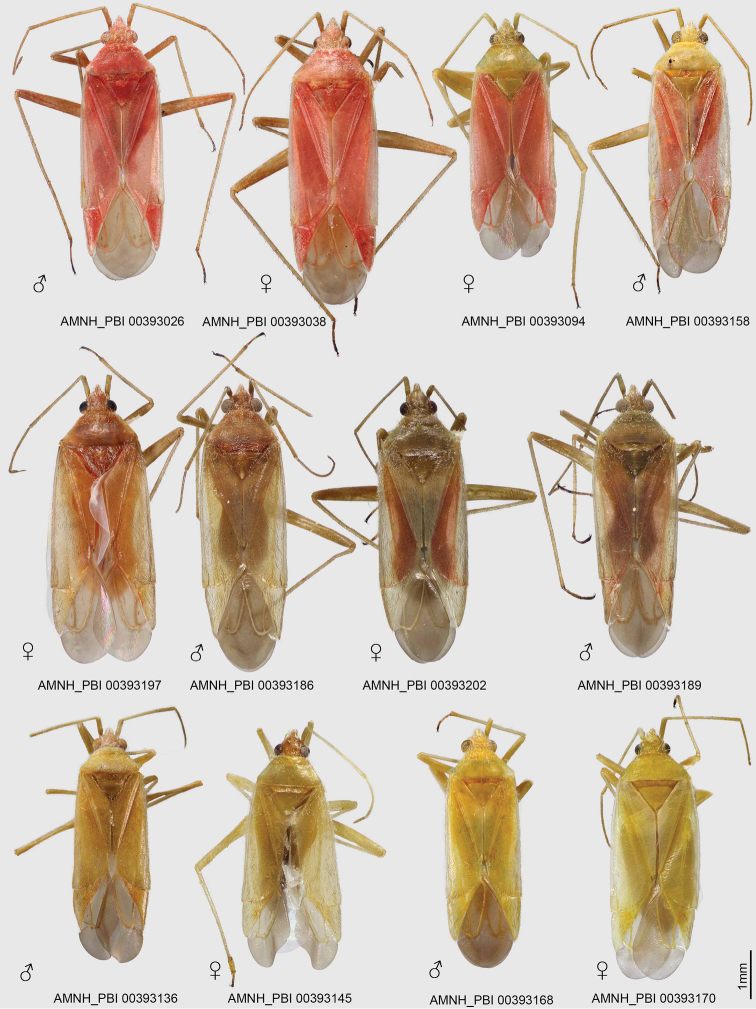
Digital habitus photographs of *Macrotylushenryi*, showing color variation.

**Figure 2.**
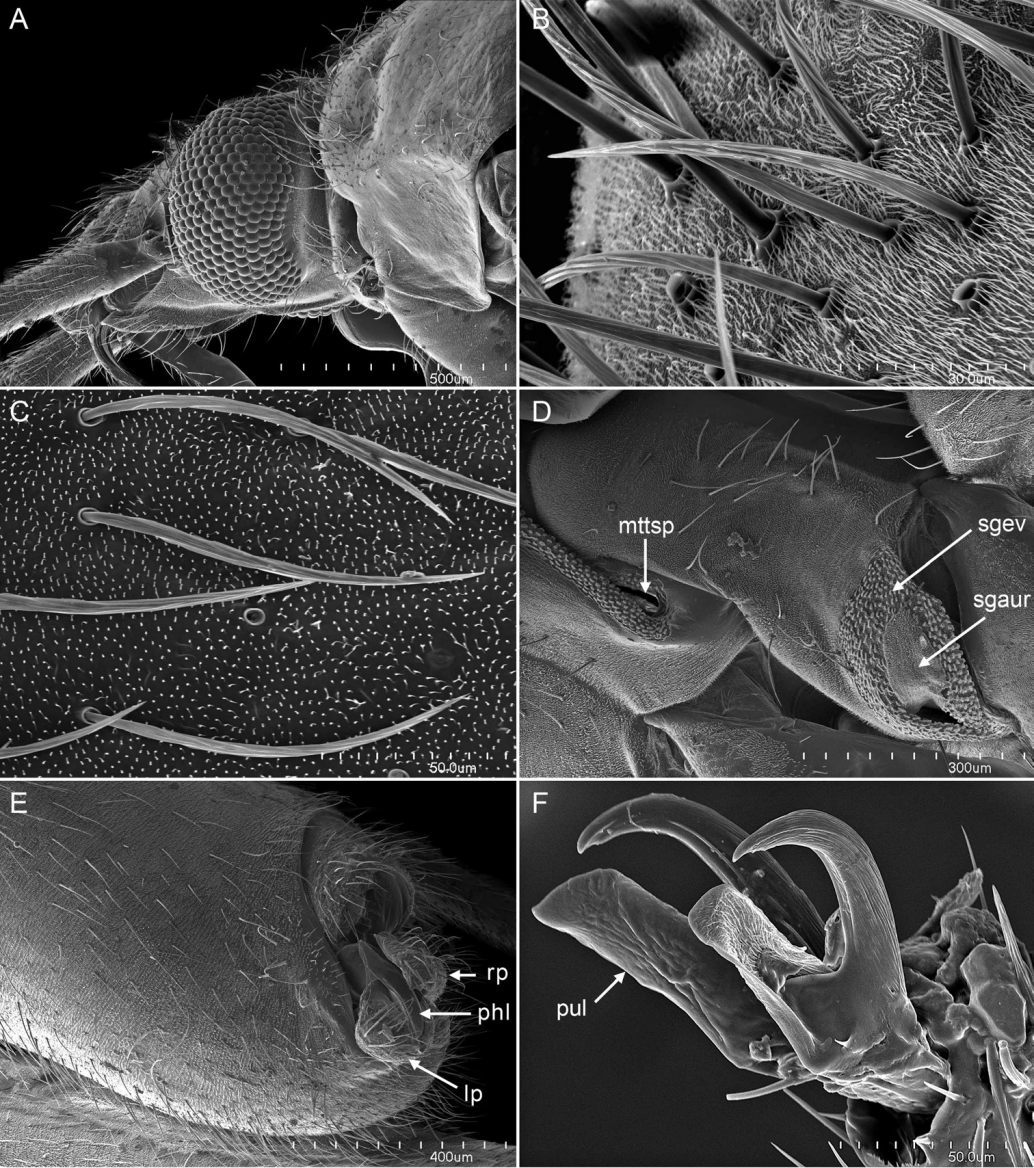
Scanning micrographs of *Macrotylushenryi* (AMNH_PBI 00393017). **A** Lateral view of head and pronotum **B** Detail of pronotal setae **C** Vestiture on hemelytron **D** Thoracic pleuron, showing metathoracic spiracle (**mttsp**), metathoracic scent-gland auricle (**sgaur**), and scent-gland evaporatory area (**sgev**) **E** Lateral view of pygophore, left paramere (**lp**), phallotheca (**phl**), and right paramere (**rp**) **F** Lateral view of pretarsus (**pul**, pulvillus).

**Figure 3.**
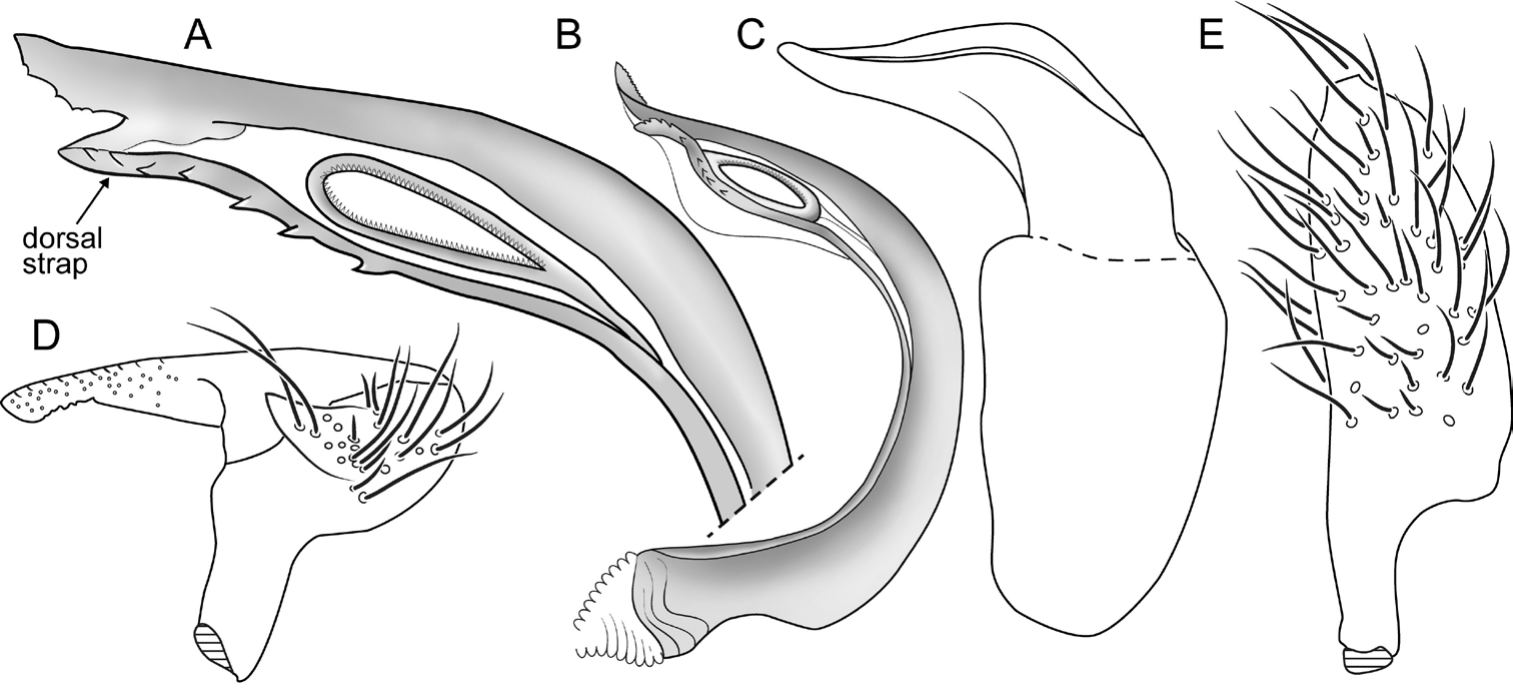
*Macrotylushenryi*, male genitalia. **A–B** Lateral view of endosoma (AMNH_PBI 00393030, AMNH_PBI 00393089) **C** Phallotheca (AMNH_PBI 00393031) **D** Lateral view of left paramere (AMNH_PBI 00393031) **E** Dorsal view of right paramere (AMNH_PBI 00393031).

**Figure 4.**
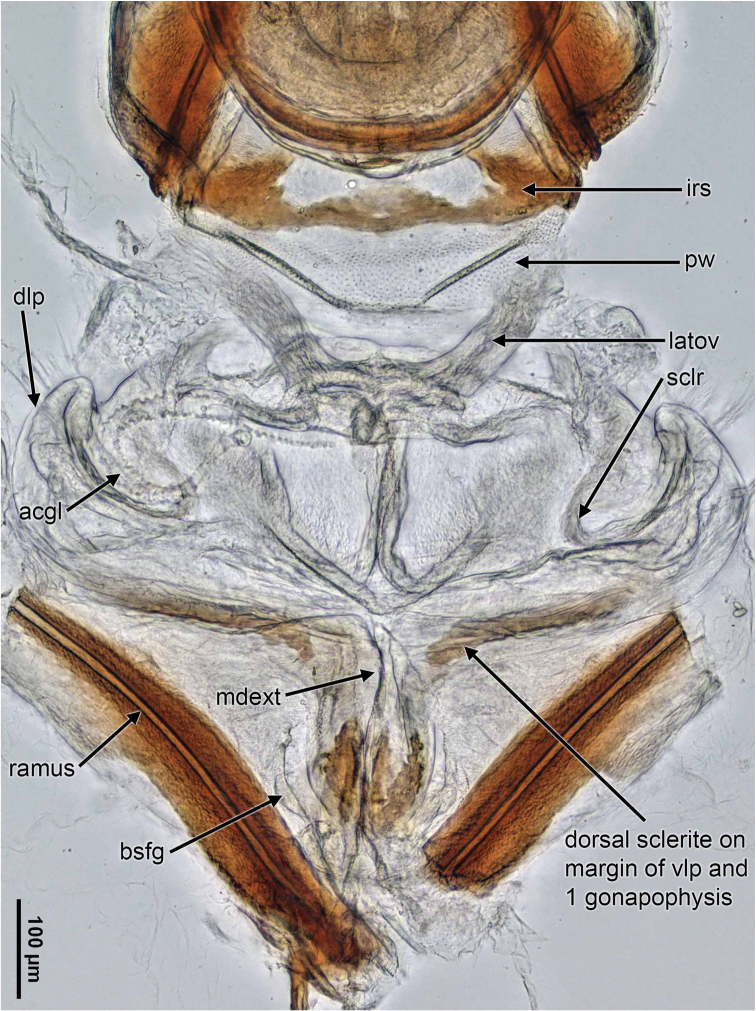
*Macrotylushenryi*, digital female genitalic images (AMNH_PBI 00393199) showing the bursa copulatrix and the bursa copulatix. Abbreviations: **acgl**, accessory gland; **bsfg**, basal sclerite of first gonapophyses; **dlp**, dorsal labiate plate; **irs**, interramal sclerites; **latov**, lateral oviduct; **mdext**, medioventral extension of ventral labiate plate; **mdscl**, medial interramal sclerite; **pw**, posterior wall; **sclr**, sclerotized ring; **vlp**, ventral labiate plate.

**Figure 5.**
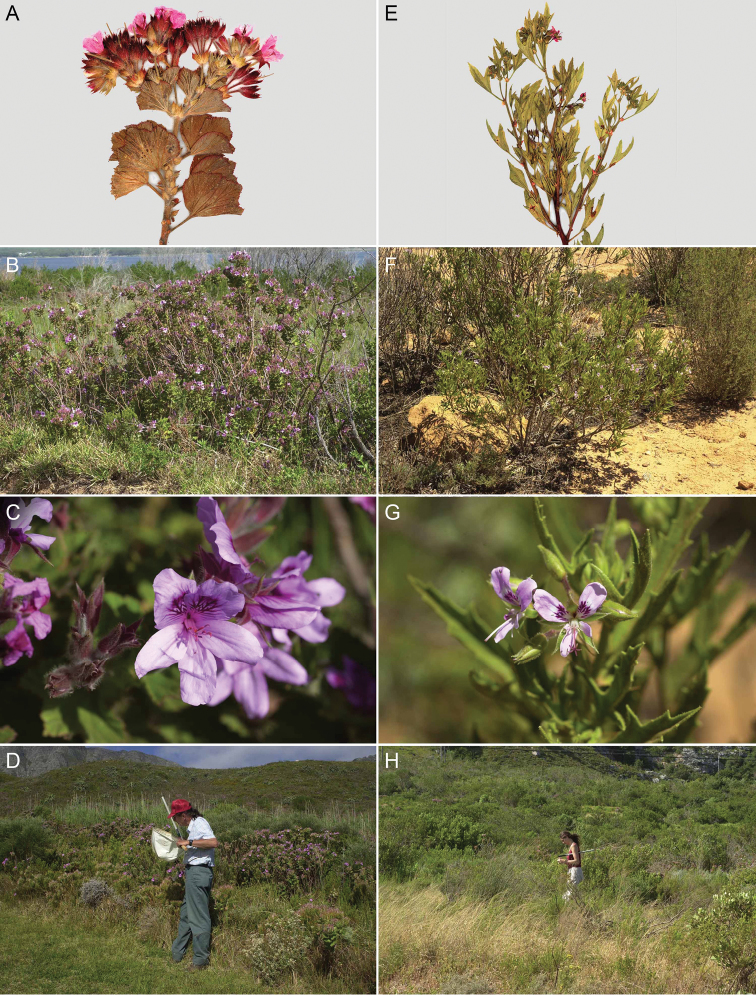
Digital photographs of living and pressed *Pelargonium* spp that are hosts of *Macrotylushenryi*, from Western Cape, South Africa **A–C**Pelargoniumcucullatum×betulinum (3.2 km E of Hermanus) **D** Randall T. Schuh collecting on P.cucullatum×betulinum (Koeel Bay, 20 km S of Strand on R44) **E–G***Pelargoniumradens* (10.5 km E of Clanwilliam, Cedarberg Range) **H** Christiane Weirauch collecting (3.2 km E of Hermanus).

**Figure 6.**
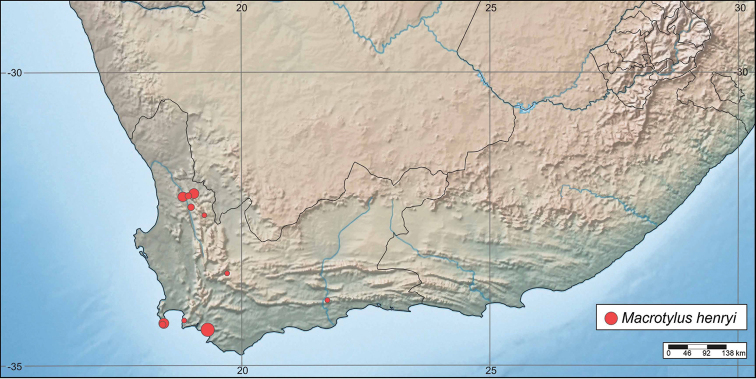
Distributions of *Macrotylushenryi* in the Western Cape province of South Africa. Dot size illustrates the relative number of specimens collected in each locality (see Table 2 for details).

Figures 1 , 2 , 3 , 4 , 5 , 6 , Tables 1 , 2

#### Diagnosis.

Placed in *Macrotylus* Fieber based on the following characteristics: The projecting clypeus, the sparsely distributed, reclining dark common setae on the dorsum (Figs 1, 2A), the pretarsus with deep tarsal claw base and long, apically free pulvillus (Fig. 2F), and the male genitalia with an elongate right paramere, deep left paramere, and the structure of endosoma (Fig. 3). Recognized among *Macrotylus* spp. by relatively large size; coloration variable, ranging from completely reddish or brownish, to a combination of brownish and reddish or yellow green and reddish, to completely yellow green; moderately projecting face; relatively narrow head, wide pronotum, long second antennal segment, and large eyes (Table 1, Figs 1, 2A); C-shaped endosoma with two sclerotized straps along margins, apical denticles, and subapical secondary gonopore (Fig. 3A–B).

Distinguished from other South African *Macrotylus* species by its larger size, vestiture type, and the structure of endosoma. *Macrotylusniger* mostly black; *M.hemizygiae*, although often yellow green as in some *M.henryi* specimens, with shining woolly setae as well as densely placed dark setae on dorsum. These two previously described South African species apparently lacking denticles seen on apex of endosoma in *M.henryi* (Fig. 3A–B, Schuh 1974: figs 264, 266). Similar to *Denticulophallus* Schuh, the other genus of Cremnorrhinina known from South Africa, based on the prominent clypeus, the enlarged free pulvilli, and the structure of endosoma (twisted, sclerotized straps, and apex with several teeth). However, *Denticulophallus* with a U-shaped endosoma with longer attenuated teeth, medial secondary gonopore, almost totally black coloration, and use of Rutaceae species as hosts (Schuh 1974, Salas in prep.).

#### Description.

***Male***. Relatively large, total length 4.85–6.26, pronotum width 1.20–1.56 (Table 1). *Coloration* (Fig. 1): Overall coloration mostly reddish, brownish, or yellow green, including appendages, or a combination of yellow green and reddish or brownish and reddish; antennae and legs similar in coloration to dorsum; membrane smoky brown.

*Surface and vestiture* (Figs 1, 2): Body surface generally with reclining common setae, broadly distributed, scattered on pronotum and scutellum, dark on hemelytra; head, pronotum, scutellum, and clavus anteriorly with sericeous woolly setae; legs also with some erect spine-like setae and tibial spines.

*Structure* (Figs 1, 2A–D, Table 1): *Head*: Moderately elongate and projecting anteriorly, relatively narrow; eyes brownish and relatively large; second antennal segment relatively long, about twice width of head; frons slightly protruding; clypeus relatively elongate and visible from above; labium surpassing hind coxae, but never reaching pygophore.

*Thorax*: Pronotum wider than long, slightly campanulate, posterior lobe weakly elevated; thoracic pleuron with sericeous setae and scattered common setae, metathoracic scent-gland evaporatory area triangular (Fig. 2D).

*Genitalia*: *Pygophore* (Fig. 2E): Occupying about 30% of abdominal length, conical, with ventral and dorsal simple and sericeous setae. *Endosoma* (Fig. 3A–B): C-shaped, with dorsal and ventral sclerotized straps seemingly adherent terminally, ventral strap wider than dorsal one; secondary gonopore subapical, moderately large relative to size of endosoma; dorsal strap with 6–9 denticles between midpoint of secondary gonopore and apex of strap; apex of ventral strap serrate and bifid, wider and extending beyond apex of dorsal one. *Phallotheca* (Figs 2E, 3C): Apical portion conical, dorsal crest well developed. *Parameres* (Figs 2E, 3D–E): Relatively large, protruding from genital aperture, with prominent setae; left paramere with posterior process long, apicoventrally serrate, and anterior process short and conical; right paramere elongate, apex blunt.

***Female***. *Coloration, surface, and vestiture* (Fig. 1): As in male, but with darker eyes. *Structure* (Fig. 1, Table 1): As in male, with similar size and body proportions. *Genitalia*: As in Fig. 4.

**Table 1.** Measurements of *Macrotylushenryi*.

	Length							Width				Ratio				
	Body	CunClyp	Head	Prono	Scut	Cun	AntSeg2	Head	Prono	Scut	IntOcDi	WH/LH	WH/WP	IOD/WH	AS2/WH	WP/LP
♂ **(N=23) Mean**	**5.49**	**3.89**	**0.57**	**0.60**	**0.52**	**0.96**	**1.67**	**0.80**	**1.37**	**0.71**	**0.35**	**1.43**	**0.59**	**0.44**	**2.09**	**2.28**
SD	0.46	0.41	0.09	0.06	0.05	0.07	0.14	0.04	0.12	0.07	0.02	0.17	0.03	0.02	0.07	0.12
Range	1.41	1.13	0.30	0.20	0.16	0.26	0.46	0.15	0.36	0.21	0.06	0.67	0.09	0.09	0.31	0.50
Min	4.85	3.43	0.43	0.51	0.46	0.82	1.46	0.74	1.20	0.63	0.32	1.15	0.54	0.39	1.93	2.04
Max	6.26	4.56	0.73	0.71	0.61	1.08	1.92	0.88	1.56	0.84	0.39	1.82	0.63	0.48	2.24	2.54
♀ **(N=23) Mean**	**5.47**	**3.96**	**0.59**	**0.63**	**0.53**	**0.89**	**1.62**	**0.79**	**1.45**	**0.74**	**0.41**	**1.35**	**0.54**	**0.52**	**2.06**	**2.32**
SD	0.48	0.37	0.07	0.05	0.05	0.08	0.11	0.05	0.11	0.06	0.02	0.12	0.02	0.02	0.06	0.09
Range	1.45	1.13	0.28	0.18	0.17	0.25	0.43	0.15	0.33	0.19	0.06	0.48	0.06	0.07	0.27	0.32
Min	4.74	3.44	0.45	0.54	0.45	0.76	1.37	0.73	1.30	0.66	0.38	1.17	0.51	0.48	1.88	2.21
Max	6.19	4.57	0.72	0.72	0.62	1.01	1.80	0.88	1.63	0.85	0.44	1.65	0.57	0.55	2.16	2.52

#### Etymology.

Named for Thomas J. Henry.

#### Hosts.

Recorded from species of *Pelargonium* L’Hér. (Geraniaceae) (Fig. 5, Table 2).

#### Distribution.

Western Cape, from near Clanwilliam to south Cape Peninsula, and east to near Mossel Bay; from sea level to ~650 m elevation (Fig. 6, Table 2).

#### Type material examined.

**Holotype: SOUTH AFRICA: Western Cape**: 3.2 km E of Hermanus, 34.40536S, 19.32737E, 33 m, 10 Nov 2003, Schuh, Cassis, Weirauch, Pelargoniumcucullatum(L.)L’Hér.×betulinum (L.) L’Hér. ex Aiton (Geraniaceae), det. K. Roux NYBG VOUCHER, 1♂ (AMNH_PBI 00393026) (PPRI).

**Paratypes: SOUTH AFRICA: Western Cape**: 3.2 km E of Hermanus, 34.40536S, 19.32737E, 33 m, 10 Nov 2003, Schuh, Cassis, Weirauch, Pelargoniumcucullatum(L.)L’Hér.×betulinum (L.) L’Hér. ex Aiton (Geraniaceae), det. K. Roux NYBG VOUCHER, 16♂ (00388606–00388621), 28♀ (00388622–00388649) (AM), 25♂ (00393000–00393021, 00393030, 00393031, 00393076), 35♀ (00393038, 00393045–00393075, 00393077–00393079) (AMNH), 1♂ (00393027), 2♀ (00393039, 00393040) (CNC), 2♂ (00393024, 00393025), 2♀ (00393036, 00393037) (PPRI), 2♂ (00393022, 00393023), 2♀ (00393034, 00393035) (SAMC), 2♂ (00388604, 00388605), 3♀ (00388650–00388652) (UNSW), 1♂ (00393028), 2♀ (00393041, 00393042) (USNM), 1♂ (00393029), 2♀ (00393043, 00393044) (ZISP). 5.6 km W of Clanwilliam on Rt 364 to Lambert’s Bay, 32.16419S, 18.83542E, 329 m, 28 Oct 2007, Schuh, Cassis, Massie, *Pelargoniumscabrum* (L.) L’Her. (Geraniaceae), det. Field ID, 8♂ (00393155–00393159, 00393167, 00393168, 00393174), 8♀ (00393160–00393164, 00393169–00393171) (AMNH), 10♂ (00387329–00387334, 00387344, 00387347–00387349), 11♀ (00387335–00387341, 00387345, 00387350–00387352) (UNSW). 10.5 km E of Clanwilliam, Cedarberg Range, 32.14699S, 18.94695E, 568 m, 29 Oct 2003, Schuh, Cassis, Weirauch, *Pelargoniumradens* H.E. Moore (Geraniaceae), det. K. Roux NYBG VOUCHER, 3♂ (00415069–00415071), 7♀ (00415078–00415084) (AMNH), 1♀ (00415086) (PPRI), 1♀ (00415085) (SAMC). 57.5 km NW of N2 on R327 beyond Herbertsdale, 33.91808S, 21.73641E, 277 m, 06 Nov 2003, Schuh, Cassis, Weirauch, *Pelargoniumscabrum* (L.) L’Her. (Geraniaceae), det. K Roux NYBG VOUCHER, 2♂ (00393129, 00393130), 1♀ (00393131) (AMNH). Nieuwoudts Pass, ~30 km N of Citrusdal on road to Algeria, 32.34585S, 18.99948E, 429 m, 27 Oct 2007, Schuh, Cassis, Massie, *Pelargoniumscabrum* (L.) L’Her. (Geraniaceae), det. K. Roux NYBG VOUCHER, 9♂ (00393135–00393141, 00393152, 00393153), 6♀ (00393144–00393148, 00393154) (AMNH), 1♂ (00393133), 1♀ (00393150) (PPRI), 1♂ (00393132), 1♀ (00393149) (SAMC), 1♂ (00393134), 1♀ (00393151) (ZISP). Table Mountain National Park, Cape Peninsula, 34.29783S, 18.44473E, 15 m, 29 Oct 2007, Schuh, Cassis, Massie, Pelargoniumcucullatum(L.)L’Hér.subsp.tabulare Volschenk (Geraniaceae), det. K. Roux NYBG VOUCHER, 17♂ (00393175–00393179, 00393185–00393193, 00393203–00393205), 8♀ (00393196–00393202, 00393206) (AMNH), 1♂ (00393182) (CNC), 1♂ (00393181), 1♀ (00393195) (PPRI), 1♂ (00393180), 1♀ (00393194) (SAMC), 4♂ (00387285–00387288), 4♀ (00387289–00387292) (UNSW), 1♂ (00393183) (USNM), 1♂ (00393184) (ZISP). Table Mountain National Park, Cape Peninsula, Circular Drive Viewpoint, 34.31722S, 18.42015E, 95 m, 29 Oct 2007, Schuh, Cassis, Massie, Pelargoniumcucullatum(L.)L’Hér.subsp.tabulare Volschenk (Geraniaceae), det. Field ID, 2♂ (00387315, 00387316) (AM), 4♂ (00387317–00387320), 2♀ (00387321, 00387322) (UNSW). ca 5 km E of de Doorns S of N1, 33.46484S, 19.72046E, 652 m, 31 Oct 2003, Schuh, Cassis, Weirauch, Wendl. (Geraniaceae), det. K. Roux NYBG VOUCHER, 1♂ (00414238), 2♀ (00414239, 00414240) (AMNH). ca 25 km E of Clanwilliam, on plains below Pakhuispas, 32.10577S, 19.0575E, 534 m, 29 Oct 2003, Schuh, Cassis, Weirauch, *Pelargoniumradens* H.E. Moore (Geraniaceae), det. K. Roux NYBG VOUCHER, 5♂ (00388653–00388656, 00388658), 4♀ (00388659–00388662) (AM), 12♂ (00393086–00393090, 00393110–00393113, 00393117, 00393118, 00415087), 19♀ (00393092–00393104, 00393120–00393125) (AMNH), 2♂ (00393083, 00393115), 2♀ (00393107, 00393127) (CNC), 1♂ (00393082), 1♀ (00393106) (PPRI), 1♂ (00393081), 1♀ (00393105) (SAMC), 2♂ (00393084, 00393116), 2♀ (00393108, 00393128) (USNM), 1♂ (00393085), 2♀ (00393109, 00393126) (ZISP).

#### Other specimens examined.

**SOUTH AFRICA: Western Cape**: 3.2 km E of Hermanus, 34.40536S, 19.32737E, 33 m, 10 Nov 2003, Schuh, Cassis, Weirauch, Pelargoniumcucullatum(L.)L’Hér.×betulinum L’Hér. ex Aiton (Geraniaceae), det. K. Roux NYBG VOUCHER, 2 nymphs (00393032, 00393033) (AMNH). 5.6 km W of Clanwilliam on Rt 364 to Lambert’s Bay, 32.16419S, 18.83542E, 329 m, 28 Oct 2007, Schuh, Cassis, Massie, *Pelargoniumscabrum* (L.) L’Her. (Geraniaceae), det. Field ID, 4 nymphs (00393165, 00393166, 00393172, 00393173) (AMNH), 4 nymphs (00387342, 00387343, 00387346, 00387353) (UNSW). 10.5 km E of Clanwilliam, Cedarberg Range, 32.14699S, 18.94695E, 568 m, 29 Oct 2003, Schuh, Cassis, Weirauch, *Pelargoniumradens* H.E. Moore (Geraniaceae), det. K. Roux NYBG VOUCHER, 6 nymps (00415072–00415077) (AMNH). Farm Dwarsrivier, Cedarberg, 32.48333S, 19.26667E, 10 Oct 2002–15 Oct 2002, D. Jacobs and M. Stillar, 1♂ (00415088) (AMNH). Koeel Bay, 20 km S of Strand on R44, 34.25187S, 18.85597E, 5 m, 11 Nov 2003, Schuh, Cassis, Weirauch, *Pelargoniumcucullatum* (L.) L’Hér. *betulinum* L’Hér. ex Aiton (Geraniaceae), det. K. Roux NYBG VOUCHER, 1♀ (00393080) (AMNH). Nieuwoudts Pass, ~30 km N of Citrusdal on road to Algeria, 32.34585S, 18.99948E, 429 m, 27 Oct 2007, Schuh, Cassis, Massie, *Pelargoniumscabrum* (L.) L’Her. (Geraniaceae), det. K. Roux NYBG VOUCHER, 2 nymphs (00393142, 00393143) (AMNH). Table Mountain National Park, Cape Peninsula, 34.29783S, 18.44473E, 15 m, 29 Oct 2007, Schuh, Cassis, Massie, Pelargoniumcucullatum(L.)L’Hér.subsp.tabulare Volschenk (Geraniaceae), det. K. Roux NYBG VOUCHER, 22 nymphs (00387293–00387314) (UNSW). Table Mountain National Park, Cape Peninsula, Circular Drive Viewpoint, 34.31722S, 18.42015E, 95 m, 29 Oct 2007, Schuh, Cassis, Massie, Pelargoniumcucullatum(L.)L’Hér.subsp.tabulare Volschenk (Geraniaceae), det. Field ID, 6 nymphs (00387323–00387328) (UNSW). ca 5 km E of de Doorns S of N1, 33.46484S, 19.72046E, 652 m, 31 Oct 2003, Schuh, Cassis, Weirauch, *Pelargoniumalternans* Wendl. (Geraniaceae), det. K. Roux NYBG VOUCHER, 1 nymph (00414241) (AMNH). ca 25 km E of Clanwilliam, on plains below Pakhuispas, 32.10577S, 19.0575E, 534 m, 29 Oct 2003, Schuh, Cassis, Weirauch, *Pelargoniumradens* H.E. Moore (Geraniaceae), det. K. Roux NYBG VOUCHER, 2 nymphs (00388657, 00388663) (AM), 3 nymphs (00393091, 00393114, 00393119) (AMNH).

## Discussion

The structure of the male genitalia shows essentially no variation across the range of specimens we include in *M.henryi*; we therefore treat all specimens under a single species in spite of the substantial variation in color and size. The endosoma is similar to that seen in many species of *Macrotylus* from the Northern Hemisphere, as well as the Cremnorrhinina more broadly, including particularly the genera *Halophylus* Schuh and Schwartz and *Pulvillophylus* Schuh and Schwartz from Australia.

Among members of the South African Phylinae, the habit of feeding on Geraniaceae is not shared with any other species, although species of Dicyphini are frequently encountered on that plant group. Schuh (1974) reported only one host plant, *Hemizygiathorncroftii* (Lamiaceae), for *Macrotylus* in South Africa. We now have documented that species of South African *Macrotylus* also feed on Geraniaceae, and that *Macrotylushenryi* feeds on five taxa of *Pelargonium* (Table 2, Fig. 5). The data also indicate that *M.henryi* shows at least generic-level host specificity.

Most species of *Macrotylus* from the Northern Hemisphere have been reported to feed on Lamiaceae, with some taxa on Rosaceae and Asteraceae (Schuh 2002–2013). The Palearctic *Macrotyluscruciatus* (Sahlberg) is the only other species of this genus that has been reported to be associated with Geraniaceae (Kerzhner 1973), but it feeds on *Geranium* L., which has worldwide distribution, whereas *Pelargonium* is native to southern Africa and Australia (van der Walt and Vorster 1983). No Cremnorrhinina known from Australia have been recorded from *Pelargonium* or from the Geraniaceae more broadly (see Schuh and Schwartz 2016).

In the Balkan Peninsula, *Cremnorrhinusbasalis* Reuter is strictly associated with ephemeral *Geraniumrotundifolium* L. (Josifov and Simov 2006) and *G.molle* L. (Simov pers. comm.), adding an additional association with Geraniaceae for the Cremnorrhinini. Schuh and Schwartz (2016) were unaware of these host associations as documented by Josifov and Simov.

*Macrotylushenryi* seems to be restricted geographically to the Western Cape (Fig. 6, Table 2). This pattern could be explained by its possible host specificity, and by the distribution of its host plant, *Pelargonium*. Nearly 90% of *Pelargonium* spp. are restricted to southern Africa, including the Republic of South Africa and adjacent parts of Namibia, with the highest species diversity found in the south-western part of South Africa (van der Walt and Vorster 1983). Nonetheless, some details of the distribution of *M.henryi* may be obscured by the specialized geographic focus of our own collecting efforts.

The distribution of *M.henryi* is similar to that seen in the well-collected *Pseudosthenarusater* Poppius and *P.brendae* Schuh and Salas (Schuh and Salas 2011).

With regard to the distribution of *Macrotylus* more broadly, there appears to be a broad disjunction on the African continent between the larger and much better known Palearctic fauna and the *Macrotylus* species from southern Africa.

**Table 2.** Host plants and localities of *Macrotylushenryi* in Western Cape, South Africa.

Host taxon	Locality	Insect specs.
** GERANIACEAE **		
* Pelargonium alternans *	Western Cape: ca. 5 km E of de Doorns S of N1	4
Pelargonium cucullatum × betulinum	Western Cape: 3.2 km E of Hermanus	129
	Western Cape: Koeel Bay, 20 km S of Strand on R44	1
Pelargonium cucullatum subsp. tabulare	Western Cape: Table Mountain National Park, Cape Peninsula	62
	Western Cape: Table Mountain National Park, Cape Peninsula, Circular Drive	14
* Pelargonium radens *	Western Cape: ca. 25 km E of Clanwilliam, on plains below Pakhuispas	60
	Western Cape: 10.5 km E of Clanwilliam, Cedarberg Range	18
* Pelargonium scabrum *	Western Cape: 5.6 km W of Clanwilliam on Rt 364 to Lambert’s Bay	45
	Western Cape: Nieuwoudts Pass, ~30 km N of Citrusdal on road to Algeria	23
	Western Cape: 57.5 km NW of N2 on R327 beyond Herbertsdale	3
Unknown	Western Cape: Farm Dwars rivier, Cedarberg	1
**TOTAL**		**360**

## Supplementary Material

XML Treatment for Macrotylus henryi

## References

[B1] Fieber FX (1858) Criterien zur generischen Theilung der Phytocoriden (Capsini auct.). Wiener Entomologische Monatschrift 2: 289–327, 329–347, 388. [1 pl.]

[B2] Josifov M Simov N (2006) Endemism among the Heteroptera on the Balkan Peninsula. Denisia 19: 879–898.

[B3] Kerzhner IM (1973) Heteroptera of the Tuvinian ASSR. Trudy Biologicheskogo Instituta Sibirskoe Otdelenie Akademiia Nauk SSSR, Novosibirsk 16: 78–92. [In Russian]

[B4] Menard KL Schuh RT Woolley JB (2014) Total-evidence phylogenetic analysis and reclassification of the Phylinae (Insecta: Heteroptera: Miridae), with the recognition of new tribes and subtribes and a redefinition of Phylini. Cladistics 30: 391–427. 10.1111/cla.12052 34788969

[B5] Schuh RT (1974) The Orthotylinae and Phylinae (Hemiptera: Miridae) of South Africa with a phylogenetic analysis of the ant-mimetic tribes for the two subfamilies for the world. Entomologica Americana 47: 1–332.

[B6] Schuh RT (2002–2013) On-line Systematic Catalog of Plant Bugs (Insecta: Heteroptera: Miridae). http://research.amnh.org/pbi/catalog/

[B7] Schuh RT Menard KL (2013) A revised classification of the Phylinae (Insecta: Heteroptera: Miridae): Arguments for the placement of genera. American Museum Novitates 3785: 1–72. 10.1206/3785.2

[B8] Schuh RT Salas R (2011) Revision of *Parapseudosthenarus* Schuh and *Pseudosthenarus* Poppius (Hemiptera: Miridae), a monophyletic group of Crotalarieae-feeding Phylinae from South Africa with discussion of hosts and distributions. African Entomology 19(3): 660–708. 10.4001/003.019.0308

[B9] Schuh RT Schwartz MD (2016) Nineteen new genera and 82 new species of Cremnorrhinina from Australia, including analyses of host relationships and distributions (Insecta: Hemiptera: Miridae: Phylinae: Cremnorrhinini). Bulletin of the American Museum of Natural History 401: 1–279. 10.1206/amnb-925-00-1-279.1

[B10] van der Walt JJA Vorster PJ (1983) Phytogeography of *Pelargonium*. Bothalia 14: 517–523. 10.4102/abc.v14i3/4.1202

